# Books and Pamphlets Received

**Published:** 1885-10-20

**Authors:** 


### BOOKS AND PAMPHLETS RECEIVED.

*Transactions of the New York State Medical Association* for the year 1884 at Utica. N.Y., July, 1885. Edited by Austin Flint, jr., M. D., of New York County. New York: D. Appleton & Co. 1885.

A neatly bound volume of 653 pages. We note that the reports and contributions are numerous, there being an addition to the usual opening addresses of 49 papers, most of which are able and interesting matter.

The following are the officers for 1884–'85: President, John P. Gray, M. D., Utica. Vice-presidents, W. H. Robb, M. D., Montgomery County; John G. Orton, M. D., Broom County; J. C. Green, M. D., Erie County; J. C. Hutchison, M. D., King's County. Recording Secretary, Caleb Green, M. D., Courtland County. Corresponding Secretary, E. D. Ferguson, M. D., Renselear County. Treasurer, John H. Hinton, M. D., New York City.

The next meeting will be held in New York City on November 17, 1885.

*A Complete Pronouncing Medical Dictionarg*, embracing the terminology of Medicine and the kindred sciences, with their signification, etymology and pronunciation; with an Appendix, comprising an explanation of the Latin terms and phrases occuring in Medicine, Anatomy, Pharmacy, etc., together with the necessary directions for writing Latin prescriptions, and a Table of Doses, etc. By Joseph Thomas, M.D., LL.D., author of the System of Pronunciation in Lippincott's "Pronouncing Gazetteer of the World," and Pronouncing Dictionary of Biography and Mythology, on the basis of Thomas' Comprehensive Medical Dictionary. Philadelphia, Pa.: J. B. Lippincott & Co.; London, 15 Russell street, Convent Garden. 1885.

We examined the above work with some care, and are pleased with it. It numbers 800 double-column octavo pages, to which is added an exceedingly useful appendix of 30 pages. The definitions are plain, condensed, and yet sufficiently full, and the vocabulary is brought down to the latest period. We pronounce it eminently useful both to the student and the practitioner.

*Poisons; their Effects and Detection;* a Manual for the use of Analytical Chemists and Experts, with an Introductory Essay on the Growth of Modern Toxocology. By Alexander Wynter Blythe, M. R. C. S. F. C. S., etc., Public Analyst for the county of Devon, and Medical Officer of Health, and Public Analyst for St. Marylebone; with Tables and Illustrations; in two volumes, aggregating 668 octavo pages.

This work is full, and brings up the "old poison lore" as well as the growth and development of the modern methods of chemically detecting poisons. Poisons are classified; statistics are given, and the connection between toxic action and chemical composition is explained, and the poisonous effects of the numerous toxic agents, organic and morganic, are delineated in a manner at once practical and interesting. The work is illustrated and ably gotten up, and must prove eminently useful and desirable.

*Insomnia and other Disorders of Sleep.* By Henry M. Lyman, A.M., M. D., Professor of Physiology and of Diseases of the Nervous System in Rush Medical College; Professor of the Theory and Practice of Medicine in the Woman's Hospital Medical College, and Physician to the Presbyterian Hospital, Chicago. Chicago, Ill.: W. F. Keener, 96 Washington street. 1885.

A work of 239 octavo pages; well written, and upon topics which have ever been regarded with strange interest by reason of the mystery that has always attended the study of the operations of the human brain and mind. We have perused the work not only with great interest but with profit. Few subjects are more interesting to the inquiring mind, especially those who are fond of psycological and physiological studies, than dreams, somnambulism, hypnoticism, mind-reading, cataleptic conditions, hypnotic clairvoyance, phenomena of spiritualism, metaphysical healing, etc., all of which, with kindred topics, are incidentally, if not directly, treated of in this work. While one may not accord with the author in all of his conclusions, he cannot fail to be interested in the perusal of this work of Professor Lyman.

